# Supplementary material for: A deep learning pipeline for mapping in situ network-level neurovascular coupling in multi-photon fluorescence microscopy
Source: eLife. 2026 Mar 24;13:RP95525. doi: 10.7554/eLife.95525 (PMC13012726; doi:10.7554/eLife.95525)
Supplement: Supplementary file 1. — Includes heart rate, breath rate, and oxygen saturation levels recorded via pulse oximetry. [file elife-95525-supp1.docx]

**Supplementary Table 1: Physiological Monitoring Data**

|  | Mean Across Subjects During Imaging |
| --- | --- |
| End-tidal pCO2 | 15.93 ± 5.07 mmHg |
| Heart rate | 318.30 ± 28.69 BPM |
| O2 Saturation | 96.30 ± 3.50 % |
| Breath Rate | 123.89 ± 12.47 BPM |
| Temperature | 37.15 ± 0.30 °C |
| Weight | 27.94 ± 6.03 g |
