# Supplementary material for: A deep learning pipeline for mapping in situ network-level neurovascular coupling in multi-photon fluorescence microscopy
Source: eLife. 2026 Mar 24;13:RP95525. doi: 10.7554/eLife.95525 (PMC13012726; doi:10.7554/eLife.95525)
Supplement: Supplementary file 2. — Detailed list of spatial and intensity transformations used for training the segmentation models. [file elife-95525-supp2.docx]

**Supplementary Table 2: Data Augmentations**

| **Category** | **Transformation** | **Parameters** |
| --- | --- | --- |
| Cropping | Random spatial cropping | Crops: 8  Crop size: 128x128x128 |
| Rotations | Random rotation 90 degrees | Max rotations: 3 |
| Mirroring | Random flipping | Axes: all |
| Rotations | Random affine transformation | Rotation restriction: 20° |
| Zoom | Random zooming | Min zoom: 0.3  Max zoom: 3 |
| Deformations | Random 3D elastic deformation | Sigma: 1 and 3 SU  Magnitude: 3-15 μm |
|  | Random grid distortion | Cells: 8  Magnitude: -0.3 to 0.3 of cell  width |
| Intensity Transformations | Random intensity shift | Offset: 0.4 SU |
|  | Random contrast adjustment | Gamma: 0.5 to 5.5 |
|  | Random histogram shift | Control points: 4 |
| Gaussian Transformation | Random Gaussian sharpening | Sigma1: 0.5 to 1 SU  Sigma2: 0.5 SU  Alpha: 10 to 30 SU |
|  | Random Gaussian smoothing | Sigma: 0.25 to 1.25 SU |
|  | Random Gaussian noise | Mean : 0 SU  STD: 0.2 SU |
| Drop pixels | Random coarse dropout | Minimum holes: 50  Maximum holes: 1000  Spatial size: 2 pixels  Fill: 0.0001 to 0.1 SU |
