# Supplementary material for: A deep learning pipeline for mapping in situ network-level neurovascular coupling in multi-photon fluorescence microscopy
Source: eLife. 2026 Mar 24;13:RP95525. doi: 10.7554/eLife.95525 (PMC13012726; doi:10.7554/eLife.95525)
Supplement: Supplementary file 3. — Comparison of model performance across different loss functions, learning rates, and dropout settings. [file elife-95525-supp3.docx]

**Supplementary Table 3: Model Performance Comparisons**

|  | **UNETR** | **UNet** | **Ilastik** |
| --- | --- | --- | --- |
| **Vessels** | | | |
| **Dice** | 0.763±0.096 | 0.790±0.088 | 0.449±0.375 |
| **Precision** | 0.811±0.089 | 0.813±0.076 | 0.377±0.347 |
| **Recall** | 0.750±0.172 | 0.797±0.165 | 0.893±0.198 |
| **Hausdorff 95%** | 13.567±12.131 | 12.116±12.144 | 38.921±35.706 |
| **Mean Surface Distance** | 1.900±1.545 | 1.737±1.622 | 1.675±1.355 |
| **Neurons** | | | |
| **Dice** | 0.712±0.062 | 0.766±0.044 | 0.487±0.196 |
| **Precision** | 0.628±0.111 | 0.698±0.091 | 0.435±0.239 |
| **Recall** | 0.841±0.053 | 0.860±0.061 | 0.814±252 |
| **Hausdorff 95%** | 23.654±19.299 | 20.340±22.67 | 50.000±26.660 |
| **Mean Surface Distance** | 1.248±0.569 | 2.409±3.901 | 4.450±7.315 |
