# Supplementary material for: A deep learning pipeline for mapping in situ network-level neurovascular coupling in multi-photon fluorescence microscopy
Source: eLife. 2026 Mar 24;13:RP95525. doi: 10.7554/eLife.95525 (PMC13012726; doi:10.7554/eLife.95525)
Supplement: Supplementary file 4. — Quantitative evaluation of UNETR, U-Net, and ilastik models based on overlap and surface distance metrics. [file elife-95525-supp4.docx]

**Supplementary Table 4: UNETR Model Generalizability for Vascular Segmentation**

|  | **THY1-ChR2-EYFP**  **(Current Study)** | **C57**  **(Different Mouse Species)** | **Fischer Rats**  **(Different Species)** |
| --- | --- | --- | --- |
| **Vessels** | | | |
| **Dice** | 0.763±0.096 | 0.663±0.124 | 0.704±0.046 |
| **Precision** | 0.811±0.089 | 0.632±0.225 | 0.723±0.117 |
| **Recall** | 0.750±0.172 | 0.766±0.052 | 0.741±0.178 |
| **Hausdorff 95%** | 13.567±12.131 | 75.548±52.978 | 65.961±46.077 |
| **Mean Surface Distance** | 1.900±1.545 | 18.482±17.918 | 22.184±22.204 |
